# Health Care for the Population Deprived of Liberty: Overcoming Challenges and Promoting Citizenship

**DOI:** 10.1590/0037-8682-0190-2024

**Published:** 2024-09-13

**Authors:** Lia Gonçalves Possuelo, Karine Zenatti Ely, Milena Mantelli Dall Soto, Eduarda Gassen Boeira, Samantha Lopes de Moraes Longo, Pauline Schwarzbold, Tiago Antônio Heringer, Ricardo Alexandre Arcênio, Julio Croda, Andreia Rosane de Moura Valim

**Affiliations:** 1 Rede Brasileira de Pesquisa em Tuberculose, Rio de Janeiro, RJ, Brasil.; 2 Universidade de Santa Cruz do Sul, Santa Cruz do Sul, RS, Brasil.; 3 Secretaria Estadual de Saúde do Rio Grande do Sul, Porto Alegre, RS, Brasil.; 4 Universidade de Santa Cruz do Sul, Programa de Pós-Graduação Stricto Sensu em Promoção da Saúde, Santa Cruz do Sul, RS, Brasil.; 5 Superintendência de Serviços Penitenciários do Rio Grande do Sul, Santa Cruz do Sul, RS, Brasil.; 6 Universidade de São Paulo, São Paulo, SP, Brasil.; 7 Universidade Federal do Mato Grosso do Sul, Departamento de Medicina, Campo Grande, MS, Brasil.; 8 Fundação Oswaldo Cruz, Campo Grande, MS, Brasil.; 9 Yale School of Public Health, Department of Epidemiology of Microbial Diseases, New Haven, CT, USA.

Dear Editor,

Since April 30, 2024, Rio Grande do Sul (RS) has suffered the biggest climate catastrophe in its history, with 467 of the 497 (93.9%) municipalities affected, and more than a hundred deaths resulting from floods. The state has been devastated by flooding, with almost two million people affected and approximately 70 thousand people in shelters^1^. Many municipalities were isolated, and bridges and roads were destroyed. There was also a lack of drinking water, food, electricity, and telecommunications. At least ten penal institutions were isolated or suffered from flooding and were severely affected. Most of them are located in the regions of the state most affected by floods, with emphasis on the *Complexo Prisional de Charqueadas* (prison colony), the *Penitenciária Modulada Estadual de Montenegro* (prison), and the *Penitenciária Estadual do Jacuí* (prison located in Jacuí)^2^. More than 1,000 people deprived of liberty (PDL) needed to be transferred, which put us on alert about the issue of rights and access to healthcare, especially regarding tuberculosis (TB), Human Immunodeficiency Virus (HIV), syphilis, and viral hepatitis. Therefore, the PDL population is highly vulnerable to these conditions. As such, in this editorial, we aimed to report the challenges in caring for PDL regarding these infections and outline initiatives and strategies to achieve equity in access to health, recognition of citizenship, and human dignity.

In Rio Grande do Sul, many intersectoral and inter-institutional actions sought to recover epidemiological indicators after the COVID-19 pandemic, including a Continuing Health Education Program aimed at workers in the prison system, including live broadcasts^3^, conversation circles^4^, and virtual competitions^5^. The project “Breaking Barriers: Prison Community in the Fight Against Tuberculosis and Hepatitis C” is currently under development. This health communication project aimed to democratize knowledge through thematic workshops for health, safety, and education workers in prison systems and Civil Society Organizations.

A series of health communication and education materials were produced for the target audience to expand the dissemination of information on techniques, methods, and political strategies for preventing and diagnosing, as well as identifying the main signs and symptoms of TB and hepatitis C. To date, more than 400 people who are part of the prison community in RS, in seven of the ten prison regions, have been mobilized by the project and trained as multipliers. However, following a climate catastrophe, the project was temporarily suspended before closure.

We highlight that the catastrophic situation in the state affects the activities of professionals in the prison system, who, in addition to working under tension, need to carry out their activities in the face of a new reality with limited or interrupted access to prison units, resulting in a lack of staff and affecting their functioning of prison units. Increased unrest among PDL is greatest during emergencies, particularly concerning logistical and operational challenges that increase the difficulties in delivering essential supplies such as food, water, and medical equipment, leading to a scarcity of resources and difficulties in providing basic services to PDL. All of these issues directly impact prison workers’ mental health, as many also suffer losses during floods, causing even more anxiety, stress, and emotional exhaustion.

Faced with a catastrophic scenario in which survival is a priority, care aimed at infectious diseases may be postponed, and the advances achieved recently may be reversed, especially regarding the prevention and continuity of treatment of these diseases.

It is worth noting that this was the third major climate event in the RS in less than 12 months: the floods of September and November 2023 that hit the penal institutions in Vale do Taquari in the central region, and now, the new flood of even greater proportions, reaching a large part of the state of Rio Grande do Sul. Disaster risk management encompasses three stages: disaster risk reduction, disaster management, and recovery^6^. Recovery includes rehabilitation and reconstruction, in which prison labor has been used with great success since the floods of September 2023. The PDL participates in the damage recovery process, including public works, such as sidewalks, schools, and general cleaning of affected cities. Moreover, they produce cleaning materials, wooden beds, and houses for rescued animals in shelters^7^. Despite their vulnerability and severe effects, the PDL has been of fundamental importance as an arm that supports reconstruction.

In times of health, economic, and climate crises, where the struggle is to access essential items, already vulnerable populations have even more pronounced differences in access. To continue advancing the fight against infectious diseases in the prison system, the sociopolitical action of all actors in the Prison Community is of fundamental importance, including prison systems, health and education professionals, Community Councils, family members, Civil Society Organizations, judiciary members, other inspection institutions, and public health and social development bodies.

In the face of climate catastrophes, beyond the walls of penal institutions, it is essential to outline strategies aligned with the intersectoral actions foreseen in the *Brasil Saudável* (“Healthy Brazil”) program, the Agenda 2030 Sustainable Development Goals of the United Nations, and the Pan American Health Organization (PAHO) initiative for the elimination of diseases in the Americas^8^. Care actions for PDL in the prison system transcend the limits of the health sector, require a broad dialogue with different government sectors, and must include public policies, such as education, prison work, reintegration, and social assistance.
