# Erratum

**DOI:** 10.1590/0037-8682-0190B-2024

**Published:** 2024-10-28

**Revista da Sociedade Brasileira de Medicina Tropical/Journal of the Brazilian Society of Tropical Medicine**

Title: Health Care for the Population Deprived of Liberty: Overcoming Challenges and Promoting Citizenship

Vol.:57 | (e00604-2024) | 2024 | doi: https://doi.org/10.1590/0037-8682-0190-2024

**Page 1/1 (Author Name)**

Julio Henrique da Rosa Croda

**Should read:**

Julio Croda

